# Endoscopic Submucosal Dissection for Gastric Subepithelial Tumors: A Single-Center Experience

**DOI:** 10.1155/2015/425469

**Published:** 2015-08-11

**Authors:** Jin Sung Lee, Gwang Ha Kim, Do Youn Park, Jong Min Yoon, Tae Wook Kim, Jong Hun Seo, Bong Eun Lee, Geun Am Song

**Affiliations:** ^1^Department of Internal Medicine, Pusan National University School of Medicine, Busan 602-739, Republic of Korea; ^2^Biomedical Research Institute, Pusan National University Hospital, Busan 602-739, Republic of Korea; ^3^Department of Pathology, Pusan National University School of Medicine, Busan 602-739, Republic of Korea

## Abstract

*Background and Aims*. Endoscopic submucosal dissection (ESD) has been accepted as a treatment modality for gastrointestinal epithelial tumors. Recently, ESD has been applied to resect subepithelial tumors (SETs) in the gastrointestinal tract, but clinical evidence on its efficacy and safety is limited. The aim of this study was to investigate the efficacy and safety of ESD for gastric SETs and to assess possible predictive factors for incomplete resection. *Patients and Methods*. Between January 2006 and December 2013, a total of 49 patients with gastric SET underwent ESD at our hospital. Clinicopathologic characteristics of patients and SETs, therapeutic outcomes, complications, and follow-up outcomes were evaluated. *Results*. The overall rates of en bloc resection and complete resection were 88% (43/49) and 84% (43/49), respectively. Complete resection rates in tumors originating from the submucosal layer were significantly higher than those in tumors originating from the muscularis propria layer (90% versus 56%, *P* = 0.028). In multivariate logistic regression analyses, tumor location (upper third: odds ratio [OR] 12.639, 95% confidence interval [CI] 1.087–146.996, *P* = 0.043) and layer of tumor origin (muscularis propria: OR 8.174, 95% CI 1.059–63.091, *P* = 0.044) were independently associated with incomplete resection. Procedure-related bleeding and perforation rates were both 4%. No recurrence was observed in patients with complete resection at a median follow-up period of 29 months (range: 7–83 months). *Conclusions*. ESD is an effective, safe, and feasible treatment for gastric SETs. The frequency of incomplete resection increases in tumors located in the upper third of the stomach and in those originating from the muscularis propria layer.

## 1. Introduction

Gastric subepithelial tumors (SETs) are mostly asymptomatic lesions with normal overlying mucosa; they are often incidentally found during endoscopic examinations (overall frequency 0.3%) [1]. Most SETs are benign, but potentially and overtly malignant lesions should not be neglected [2]. According to the position of the American Gastrointestinal Association Institute, patients with SETs < 3 cm can be followed up with periodic endoscopy or endoscopic ultrasonography (EUS) [3]. However, this approach involves issues related to patient compliance, cost-effectiveness, and the risk associated with repeated endoscopic procedures and delayed diagnosis of malignancy [4, 5].

The differential diagnosis of SETs is not easy and includes nonneoplastic lesions, benign neoplasms, and, potentially, overtly malignant tumors [6]. The nature of the SETs cannot be determined based only on endoscopic findings. EUS is an important diagnostic method for the differential diagnosis of various SETs, and it provides valuable information on SETs including their exact size, echo patterns, and layer of origin [7, 8]; however, diagnostic accuracy for gastric SETs is not satisfactory [9]. Therefore, histologic examination is necessary for accurate diagnosis. However, standard endoscopic forceps biopsies [1], bite-on-bite technique using standard biopsy forceps or large-capacity (“jumbo”) forceps [10, 11] and EUS-assisted tissue sampling methods, including EUS-guided fine-needle aspiration or EUS-guided Tru-Cut biopsy [12–15], have reported disappointing results. Other more invasive methods such as biopsy of the SET after incision or partial removal of the overlying mucosa have been proposed, but data are limited on their effectiveness and safety profiles [16, 17].

Endoscopic submucosal dissection (ESD) has been accepted worldwide as a treatment modality with clinical evidence for gastrointestinal epithelial tumors. The ESD procedure is composed of circumferential mucosal incision and dissection of the connective tissue just below the tumor under direct visualization. If a resected lesion has a sufficient lateral margin from the circumferential incision and a sufficient vertical margin through subtumoral dissection, complete resection can theoretically be accomplished, implying that ESD can be applied to SETs [18]. Recently, several studies have reported successful ESD for gastric SETs that are located in the muscularis propria (MP) layer [19–22]. However, evidence on the utility and safety of ESD in the resection of gastric SETs, especially SETs originating from the submucosal (SM) layer, is limited. Therefore, we aimed to investigate the efficacy and safety of ESD for gastric SETs located in the MP layer as well as in the SM layer and to assess possible predictive factors for incomplete resection.

## 2. Patients and Methods

### 2.1. Patients

**Figure 1:**
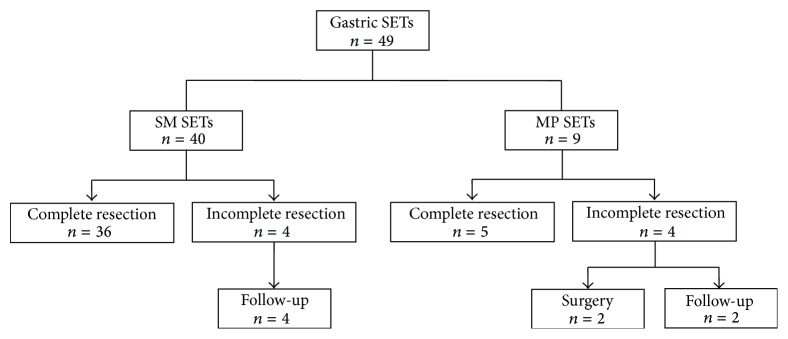
Flowchart of patients included in the study. SETs: subepithelial tumors; SM: submucosa; MP: muscularis propria.

We retrospectively analyzed our database of all patients who underwent ESD at the Pusan National University Hospital (Busan, Korea) between January 2006 and December 2013. We identified a total of 49 patients who underwent ESD for gastric SETs (Figure 1). All patients underwent EUS before the procedure and agreed to undergo ESD after explanation of the risks and benefits, including complications of ESD and the possible necessity for additional surgical treatment. Patients with well-differentiated neuroendocrine tumor (WDNET) underwent abdominal computed tomography (CT) to determine the presence of lymph node or distant metastases before ESD. Written informed consent was obtained from all patients before ESD, and the study protocol was reviewed and approved by the Institutional Review Board of Pusan National University Hospital.

### 2.2. Endoscopic Ultrasonography

EUS was performed with a radial-scanning 20 MHz catheter probe (UM3D-DP20-25R, Olympus Co., Ltd., Tokyo, Japan) or a radial-scanning ultrasonic endoscope (GF-UM2000, Olympus Co., Ltd.) at 7.5 or 12 MHz to determine the layer of origin and exact size of the tumor. SETs were classified as (1) originating from the SM layer or (2) originating from the MP layer.

### 2.3. Endoscopic Submucosal Dissection

**Figure 2:**
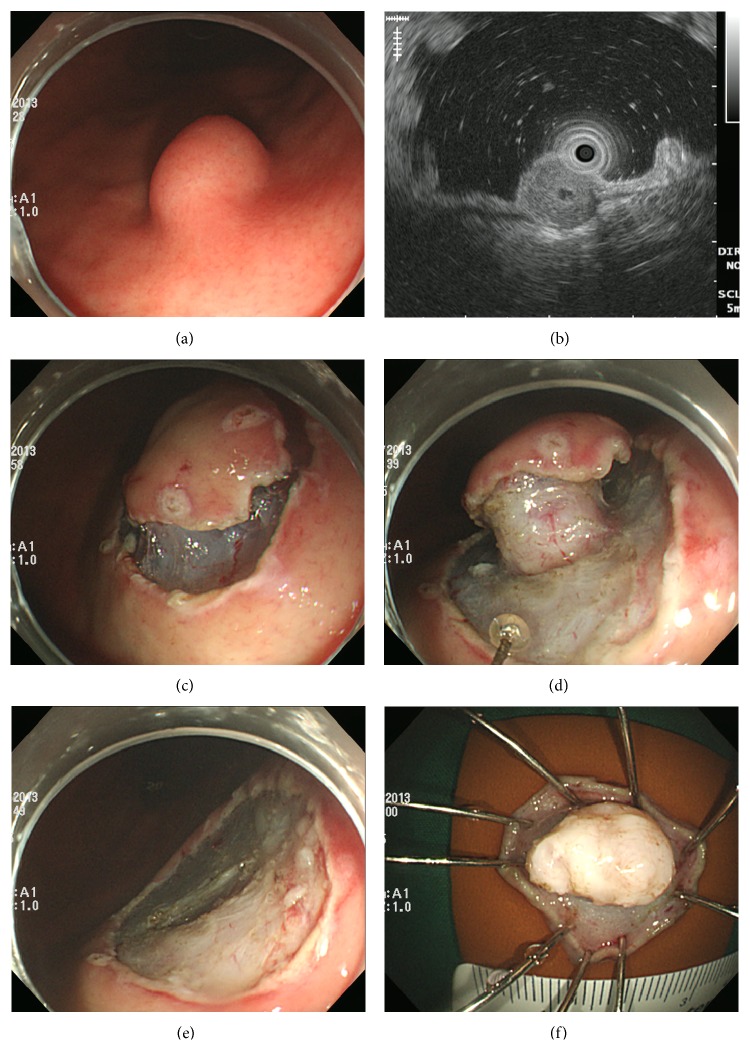
Endoscopic submucosal dissection of a gastric GIST. (a) A subepithelial tumor is observed in the middle third of the stomach. (b) The tumor originates from the muscularis propria layer on EUS. (c) After marking, circumferential precutting is performed. (d) Submucosal dissection of the tumor is performed using an IT knife. (e) The lesion is removed completely. (f) Inner surface of the resected specimen.

ESD procedures were performed by 2 experienced endoscopists (G. H. Kim, G. A. Song), using a single-channel endoscope (GIF-H260 or GIF-Q260; Olympus Co., Ltd.). Procedures were performed with the patient under conscious sedation with cardiorespiratory monitoring. For sedation, midazolam 5–10 mg and meperidine 25 mg were administered intravenously. Propofol was administered as needed during the procedure. First, dots marking the incision were placed 2 mm beyond the tumor margins with argon plasma coagulation. A saline solution (0.9% saline with a small amount of epinephrine and indigo carmine) was then injected into the SM layer around the lesion, and a circumferential incision was made with a flex knife (Fixed Flexible Snare, Kachu Technology, Seoul, Korea) or insulation-tipped (IT) knife (ESD-Knife, MTW Endoskopie, Wesel, Germany). Then, the normal tissue just beneath the lesion was directly dissected using the flex or IT knife (Figure 2). If necessary during the procedure, the saline injection was repeated and endoscopic hemostasis was achieved. A high-frequency electrosurgical current generator (Erbotom VIO 300D; ERBE, Tübingen, Germany) was used during marking, mucosal incision, subtumoral dissection, and hemostasis.

### 2.4. Histopathological Evaluation

Paraffin-embedded resected specimens were sectioned and stained with hematoxylin and eosin. If needed, additional immunohistochemical staining for c-kit (CD117), DOG-1, CD34, desmin, smooth muscle antigen (SMA), or S-100 protein was performed to differentiate tumors of mesenchymal origin. Mesenchymal lesions that stained positive for SMA and desmin were diagnosed as leiomyomas. Lesions that stained positive for c-kit or DOG-1 and CD34 were diagnosed as gastrointestinal stromal tumors (GISTs). Lesions that stained positive for S-100 were diagnosed as neurogenic tumors. The malignant potential of GISTs was categorized based on tumor size and mitotic counts per 50 high-power fields as per the consensus meeting report from the National Institutes of Health [23].

### 2.5. Follow-Up

All patients who were treated with ESD underwent postprocedural chest radiography and second-look endoscopy on the following day to detect any perforation or bleeding. Proton pump inhibitors and sucralfate were administered to relieve pain, prevent procedure-related bleeding, and promote ulcer healing. Patients without serious symptoms or adverse events were permitted to start food intake the day after the procedure and were discharged within 3-4 days.

In cases of complete resection, follow-up endoscopy was conducted 6 months after ESD and annually thereafter. In cases with complete resection for WDNET or GIST, abdominal CT, chest radiography, and laboratory measurements of tumor markers were performed 6 months after ESD and annually thereafter. In cases of incomplete resection for GIST, an additional surgery was recommended for curative resection. However, for patients who refused surgical operation, follow-up endoscopy and abdominal CT were conducted 1-2 months and 4–6 months after ESD.

### 2.6. Outcome Parameters

The primary outcome parameter was the success of the endoscopic resection, such as the rates of en bloc resection and complete resection. The secondary outcome parameters were procedure time, procedure-related complications, and local recurrence rate. En bloc resection was defined as a resection in a single piece. Complete resection was defined as successful en block resection, with no apparent residual tumor at the resection site (assessed macroscopically by the endoscopist) and with negative margins on pathologic examination.

Procedure time was defined as the time from the start of marking to complete removal of the tumor. Procedure-related bleeding was defined as (1) bleeding shown via endoscopic evaluation within 24 hours, (2) clinical evidence of melena or hematemesis, or (3) massive bleeding requiring transfusion [24]. Bleeding occurring during the ESD procedure that was treated endoscopically was not regarded as procedure-related bleeding. Perforation was endoscopically diagnosed during the procedure or by the presence of free air on plain chest radiography after ESD.

### 2.7. Statistical Analysis

Variables were expressed as medians or range and simple proportions. For univariate analyses, continuous variables were analyzed using the Mann-Whitney *U* test, and categorical variables were analyzed using the *χ* ^2^ test or Fisher's exact test. Multiple logistic regression analyses with forward stepwise regression were used to identify possible covariates as significant predictors of incomplete resection. Significant factors in the univariate analysis, defined as *P* < 0.05, or factors with clinical correlation were included in the multivariate model to assess independent factors for incomplete resection. Multivariate comparisons were expressed as odds ratios (OR) with 95% confidence intervals (CI). A *P* value of < 0.05 was considered statistically significant. The statistical calculations were performed with SPSS version 21.0 for Windows software (SPSS Inc., Chicago, IL, USA).

## 3. Results

### 3.1. Clinicopathologic Characteristics of Patients and Subepithelial Tumors

Clinicopathologic characteristics of the patients and tumors are summarized in Table 1. The patients included 17 men and 32 women with a median age of 58 years (range: 26–71 years). At the index endoscopy, 19 tumors were located in the upper third of the stomach, 11 in the middle third, and 19 in the lower third. On EUS, 40 tumors (82%) were located in the SM layer and 9 (18%) in the MP layer. Median tumor size was 9 mm (range: 4–80 mm). The tumor sizes were ≤ 20 mm in 42 lesions (86%) and > 20 mm in 7 (14%). Pathologic diagnosis was obtained in all cases: WDNET (*n* = 14), inflammatory fibrinoid polyp (*n* = 11), GIST (*n* = 7), ectopic pancreas (*n* = 4), lipoma (*n* = 4), granular cell tumor (*n* = 3), mucosa-associated lymphoid tissue lymphoma (*n* = 2), leiomyoma (*n* = 1), schwannoma (*n* = 1), fibroma (*n* = 1), and duplication cyst (*n* = 1).

### 3.2. Outcomes of Endoscopic Submucosal Dissection

Table 2 shows the outcomes for ESD of the gastric SETs. En bloc resection rate was 88% (43/49), and piecemeal resection rate was 12% (6/49). Of the en bloc resected lesions, 2 had positive vertical margins: one was schwannoma and the other was WDNET. Therefore, the complete resection rate was 84% (41/49). The median procedure time was 18 minutes (range: 6–140 minutes).

According to the layer of tumor origin, en bloc resection and complete resection rates in tumors originating from the SM layer were significantly higher than those in tumors originating from the MP layer (95% versus 56%, *P* = 0.007, and 90% versus 56%, *P* = 0.028, resp.; Table 2). The median procedure time in tumors originating from the SM layer was significantly longer than that in tumors originating from the MP layer (14.5 min versus 27.0 min, *P* = 0.003).

Procedure-related bleeding and perforation rates were both 4% (Table 2). Bleeding was observed in 2 cases (on the second day after ESD in a tumor originating from the MP layer and on the eighth day after ESD in a tumor originating from the SM layer, resp.), but all bleeding was managed successfully with endoscopic hemostasis. Perforation was noticed during the procedure in 2 cases: a tumor originating from the SM layer (diagnosed as granular cell tumor) and a tumor originating from the MP layer (diagnosed as GIST). The perforation was completely closed using hemoclips. They were treated nonoperatively with nothing by mouth and antibiotics for 3 days. There was no difference in bleeding and perforation rates between tumors originating from the SM and MP layers (both *P* = 0.337).

### 3.3. Factors Associated with Incomplete Resection

In univariate analyses, tumor location and layer of tumor origin were each significantly associated with incomplete resection (*P* = 0.022 and *P* = 0.028, resp.; Table 3). Tumor size and directional distribution of tumor were not related to incomplete resection (*P* = 0.320 and *P* = 0.997, resp.). In multivariate logistic regression analyses, tumor location (upper third: OR 12.639, 95% CI 1.087–146.996, *P* = 0.043) and layer of tumor origin (MP: OR 8.174, 95% CI 1.059–63.091, *P* = 0.044) were independently associated with incomplete resection (Table 4).

### 3.4. Follow-Up and Operation

Of the 41 patients in whom tumors were completely resected with ESD, 29 were followed up for ≥ 6 months. During the median follow-up period of 29 months (range: 7–83 months), no tumor recurrence was detected.

Of 8 incompletely resected tumors, 4 were GISTs, 2 were WDNETs, 1 was granular cell tumor, and 1 was schwannoma (Table 5). In 2 cases with incompletely resected GISTs, remnant tumor tissue was not seen macroscopically after endoscopic resection, and pathologic results indicated very low risk. Therefore, they were followed up without additional surgical resection and were tumor-free at 41 months and 53 months after ESD, respectively. In the other 2 cases with incompletely resected GISTs, macroscopically remnant tumor was present after endoscopic resection and pathologic results indicated intermediate risk and high risk, respectively. Therefore, they underwent additional laparoscopic surgical resection, and there has been no recurrence until the present. In 2 cases of incompletely resected WDNETs, remnant tumor tissue was not seen macroscopically after ESD; thus the patients were recommended to be followed up without additional surgical resection; one was lost to follow-up and the other had no recurrence for 40 months.

## 4. Discussion

The clinical application of ESD for gastric SETs has increased in recent years, but most studies have been conducted on a smaller scale and have primarily been concerned with the technical feasibility per se [5, 20, 21, 25]. Furthermore, only a few studies have included gastric SETs originating from both the SM and MP layers or have investigated factors related to complete resection. In the present study, the technical outcomes of ESD for gastric SETs were excellent, but they were influenced significantly by the tumor location and layer of tumor origin. During the relatively long-term follow-up period (median: 29 months), recurrence did not occur. These results provide important information to assist endoscopists in assessing the potential difficulties and safety in performing ESD for gastric SETs before undertaking the procedure.

In the present study, we achieved en bloc resection and complete resection rates of 88% and 84%, respectively, for ESD of gastric SETs. According to the layer of tumor origin, complete resection rates in tumors originating from the SM and MP layers were 90% and 56%, respectively. Four studies akin to the current study reported similar rates of successful en bloc resection for SETs that originated from the SM (79%–100%) and MP layers (61%–68%) [5, 26–28]. On the other hand, another recent study on ESD for 144 gastric SETs originating in the MP layer showed a very high complete resection rate (92%) [21]. In this previous study, en bloc resection was achieved in 134 tumors, and all en bloc resected cases were confirmed as complete resection. Naturally, some differences in the results probably are related to the retrospective design, the different inclusion criteria used by the available studies, and the small number of evaluated patients. In addition, even though en bloc resection was achieved, if the normal tissue covering the tumor was damaged in some portion, the final pathologic result would be incomplete resection. Therefore, this high complete resection rate might have been biased by any process during the preparation of pathologic specimens.

In a recent study analyzing factors related to the rate of complete resection following ESD for gastric SETs, the area connected to the MP layer was a factor related to complete resection, whereas the tumor size and location were not [5]. In another similar study, a positive rolling sign and tumor size ≤ 2 cm were related to complete resection [20]. In the present study, which included more SETs originating from the SM layer, univariate analyses showed that incomplete resection was associated with tumor location and layer of tumor origin, and tumor size was not associated with incomplete resection. In the multivariate analyses, tumor location in the upper third of the stomach (OR 12.639) and origination from the MP layer (OR 8.327) were significant predictors of incomplete resection. During ESD for gastric tumors in the upper third of the stomach, it is very difficult for endoscopists to permit the knife to encroach into the subtumoral layer and to maintain control of the direction and depth well according to the dissection plan [29]. In fact, several studies regarding ESD for early gastric cancer have reported an increased incomplete resection rate when the lesion was located in the upper third of the stomach [29–31]. This could also explain the higher incomplete resection rate in SETs located in the upper third of the stomach in the present study. Considering the structure of the gastric wall layer, it is natural that incomplete resection would be increased in tumors originating from the MP layer. If the tumor originated from the SM layer, the underlying MP layer just beneath the tumor would help provide support during ESD for SETs. As a result, it is not difficult to resect the tumor completely.

On the other hand, if the tumor has originated from the MP layer, the underlying structure beneath the tumor consists of only the very thin serosal layer with or without the compressed MP layer. Thus, to resect the tumor completely, it is necessary to cut the connecting muscle fibers without any damage to normal tissues covering the tumor, as the presence of the normal tissue is very important to confirm the complete resection on pathology. However, it is not easy to cut the connecting muscle fibers in the case of tumors having wide or tight attachment with surrounding muscle fibers. At the same time, this situation raised the risk of perforation, especially in the beginning period of ESD for SETs; this could cause endoscopists to resect the tumor without securing adequate margins. In fact, ESD for 2 tumors originating from the MP layer was performed in the early term of our study, and they were not resected en bloc. The other 2 tumors originating from the MP layer were macroscopically incompletely resected because of their tight and wide attachment with surrounding muscle fibers.

Perforation is not a rare complication during ESD. In the present study, perforations occurred in 2 patients (4%), and they were successfully closed by applying hemoclips without surgery. Considering ESD only for tumors originating from the MP layer, the perforation rate was 13%. Both perforations occurred in the fundus, likely because the fundus has a thin wall and getting the knife parallel to gastric wall beneath the tumor in fundic tumors is difficult to achieve; this finding is similar to previous studies [21, 32]. Bleeding occurred in 2 patients (4%), which is in accordance with the bleeding rate (5%) in our previous study about ESD for early gastric cancer [24]; bleeding was managed successfully with endoscopic hemostasis. The procedure-related complication rate in the present study is also consistent with previous studies [5, 20, 21, 27].

To increase the complete resection rate for gastric SETs and overcome problems with procedure-related perforation, endoscopic submucosal tunnel dissection [33, 34] and endoscopic full-thickness resection with or without laparoscopic assistance [35–37] have recently been suggested. However, studies on the use of such techniques are still limited to case reports and small, retrospective, or pilot series. Therefore, it should be emphasized that further studies are needed to show the feasibility of these types for tumor resection, particularly with regard to a safe and complete resection.

Gastric neuroendocrine tumors show a broad range of clinical behaviors, and their malignant features are associated with the size and depth of invasion. When the tumor size is ≤ 1 cm and the depth of invasion is limited to the SM layer, there is minimal risk of lymph node metastasis and endoscopic resection is considered as appropriate management [38]. In the present study, 14 cases of WDNET were treated by ESD. The complete resection rate was 86% (12/14); in two cases with incomplete resection, there was no macroscopic remnant tissue. In 11 cases that were followed up, no recurrence was detected during the median follow-up period of 36 months (range: 12–60 months).

Although the present study involved a relatively large number of patients, demonstrated a favorable long-term prognosis associated with ESD, and provided robust evidence that ESD is effective and safe, there are several limitations. First, there may have been potential selection or information biases resulting from the retrospective nature of the study. Although most results of ESD were prospectively collected by the endoscopists at the time of the endoscopy, patients were selected for ESD according to the clinical opinions and decisions of the medical doctors and patients' needs. Second, the number of SETs originating from the MP layer was small, compared to the number of SETs originating from the SM layer. This limitation might be due to the fact that the most common mesenchymal tumor of the stomach is GIST and that the treatment of choice for GIST is surgery. In our previous studies [39, 40], it was possible to differentiate GIST from non-GIST mesenchymal tumors such as schwannoma or leiomyoma to some degree using EUS. Therefore, there is some tendency for medical doctors to recommend surgery for patients with suspicious GIST rather than follow-up and to regularly follow up those with suspicious non-GIST mesenchymal tumors.

In conclusion, the present results showed that ESD is an effective, safe, and feasible treatment for gastric SETs. The frequency of incomplete resection increases in tumors located in the upper third of the stomach or originating from the MP layer. Therefore, gastric SETs originating from the SM layer are ideal candidates for ESD. However, for SETs originating from the MP layer, it is necessary to know the limitations of ESD and to choose adequate cases with the possibility of high complete resection. Further prospective multicenter studies, including more cases of SETs originating from the MP layer, will give more useful information regarding ESD for gastric SETs.

## Figures and Tables

**Table 1:** Characteristics of the patients and subepithelial tumors.

Median age, years (range)	58 (26–71)
Gender, *n* (%)	
Male	17 (35)
Female	32 (65)
Tumor location, *n* (%)	
Upper third	19 (39)
Middle third	11 (22)
Lower third	19 (39)
Directional distribution, *n* (%)	
Anterior wall	12 (24)
Posterior wall	17 (35)
Lesser curvature	7 (14)
Greater curvature	13 (27)
Tumor size, *n* (%)	
≤20 mm	42 (86)
>20 mm	7 (14)
Layer of tumor origin, *n* (%)	
Submucosa	40 (82)
Muscularis propria	9 (18)
Pathological diagnosis, *n* (%)	
Well-differentiated neuroendocrine tumor	14 (29)
Inflammatory fibrinoid polyp	11 (22)
Gastrointestinal stromal tumor	7 (14)
Ectopic pancreas	4 (8)
Lipoma	4 (8)
Granular cell tumor	3 (6)
MALT lymphoma	2 (4)
Leiomyoma	1 (2)
Schwannoma	1 (2)
Fibroma	1 (2)
Duplication cyst	1 (2)

MALT: mucosa-associated lymphoid tissue.

**Table 2:** Therapeutic outcomes of endoscopic submucosal dissection for gastric subepithelial tumors according to the layer of tumor origin.

	Total	SM	MP	*P* value
	(*n* = 49)	(*n* = 40)	(*n* = 9)	
En bloc resection, *n* (%)	43 (88)	38 (95)	5 (56)	0.007
Complete resection, *n* (%)	41 (84)	36 (90)	5 (56)	0.028
Cause for incomplete resection, *n* (%)				
Lateral involvement	3 (6)	1 (3)	2 (22)	0.083
Vertical involvement	8 (16)	4 (10)	4 (44)	0.028
Median procedure time, min (range)	18.0 (6.0–140.0)	14.5 (6.0–65.0)	27.0 (13.0–140.0)	0.003
Procedure-related complications, *n* (%)				
Bleeding	2 (4)	1 (3)	1 (11)	0.337
Perforation	2 (4)	1 (3)	1 (11)	0.337

SM: submucosa; MP: muscularis propria.

**Table 3:** Univariate analyses for predictive factors of incomplete resection with endoscopic submucosal dissection for gastric subepithelial tumors.

	Complete resection (*n* = 41)	Incomplete resection (*n* = 8)	*P* value
Age, *n* (%)			0.710
≤60 years	24 (86)	4 (14)	
>60 years	17 (81)	4 (19)	
Gender, *n* (%)			0.423
Male	13 (76)	4 (24)	
Female	28 (88)	4 (12)	
Tumor location, *n* (%)			0.022
Upper third	13 (68)	6 (32)	
Middle third	9 (82)	2 (18)	
Lower third	19 (100)	0 (0)	
Directional distribution, *n* (%)			0.997
Anterior wall	10 (83)	2 (17)	
Posterior wall	14 (82)	3 (18)	
Lesser curvature	6 (86)	1 (14)	
Greater curvature	11 (85)	2 (15)	
Tumor size, *n* (%)			0.320
≤20 mm	36 (86)	6 (14)	
>20 mm	5 (71)	2 (29)	
Layer of tumor, *n* (%)			0.028
Submucosa	36 (90)	4 (10)	
Muscularis propria	5 (56)	4 (44)	

**Table 4:** Multivariate analyses for predictive factors of incomplete resection with endoscopic submucosal dissection for gastric subepithelial tumors.

Variables	Odds ratio	95% confidence intervals	*P* value
Tumor location (upper third)	12.639	1.087–146.996	0.043

Tumor size (>20 mm)	5.740	0.270–121.838	0.262

Layer of tumor origin (muscularis propria)	8.174	1.059–63.091	0.044

**Table 5:** Clinicopathologic characteristics of gastric subepithelial tumors incompletely resected by endoscopic submucosal dissection.

Patient number	Gender/ age	Tumor location	Tumor size (mm)	Layer of tumor origin	Pathologic diagnosis	Additional surgery	Follow-up period (mo)	Recurrence
1	M/47	Upper	8	SM	WDNET	No	—	—
2	M/71	Upper	7	SM	WDNET	No	40	No
3	F/52	Upper	12	SM	GCT	No	9	No
4	F/62	Upper	4	SM	Schwannoma	No	—	—
5	M/46	Middle	10	MP	GIST	No	41	No
6	M/49	Middle	32	MP	GIST	Yes	25	No
7	M/61	Upper	5	MP	GIST	No	53	No
8	F/69	Upper	44	MP	GIST	Yes	33	No

SM: submucosa; MP: muscularis propria; WDNET: well-differentiated neuroendocrine tumor; GCT: granular cell tumor; GIST: gastrointestinal stromal tumor.
